# Inflammatory myofibroblastic tumour of an unusual presentation in the uterine cervix: a case report

**DOI:** 10.1186/s12957-021-02438-5

**Published:** 2021-11-20

**Authors:** Alfonso López de Sa, Alejandro Pascual, Javier Garcia Santos, Ramiro Mendez, Monica Bellon, Mar Ramirez, Fatima Matute, Cristina del Arco, Aránzazu Manzano, Pluvio Coronado, Antonio Casado, Gloria Marquina

**Affiliations:** 1 Department of Medical Oncology, Hospital Clinico san Carlos, Department of Medicine, School of Medicine, Universidad Complutense de Madrid (UCM), IdISSC, Madrid, Spain; 2 Department of Pathology, Hospital Universitario Clinico San Carlos, Madrid, Spain; 3 Gynaecologic Oncology Unit, Hospital Clinico san Carlos, Department of Obstetrics and Gynaecology, School of Medicine, Universidad Complutense de Madrid (UCM), IdISSC, Madrid, Spain; 4 Department of Radiology, Hospital Clinico san Carlos, Department of Radiology and Physics Medicine, School of Medicine, Universidad Complutense de Madrid (UCM), IdISSC, Madrid, Spain

**Keywords:** Inflammatory myofibroblastic tumour, Soft tissue sarcoma, Mesenquimal neoplasia, Gynaecologic tumour, Cervical tumour

## Abstract

**Background:**

Inflammatory myofibroblastic tumour is an infrequent mesenchymal neoplasia of unknown aetiology and variable behaviour, ranging from rather benign lesions to locally aggressive and even metastatic disease. Its presence has been described in almost all organs; however, its location in the female genital tract has rarely been reported.

**Case presentation:**

We present the case of a 47-year-old female, who was studied in our institution for a recent medical history of several weeks of dyspareunia and abdominal pain. She underwent pertinent studies including ultrasonography and CT scan. Under suspicion of degenerated leiomyoma, a total hysterectomy was performed. Unexpectedly, the pathological study of the surgical specimen showed very few tumour cells with focal fusiform morphology surrounded by an abundant inflammatory infiltrate; a thorough immunohistochemistry study lead to myofibroblastic tumour of the cervix diagnosis. A PET-CT scan did not show metastatic disease. The patient did not undergo any adjuvant treatment, and she is currently on surveillance with no evidence of disease relapse.

**Conclusions:**

Inflammatory myofibroblastic tumour remains a rare entity yet to be fully elucidated. The diagnosis is based on pathological study due to the lack of typical clinical manifestations and typical radiological images. Surgical resection is the most frequent treatment, whereas chemotherapy and radiotherapy are restricted to locally advanced or metastatic disease. Tirosine kinase inhibitor crizotinib has shown promising results especially in tumours harbouring ALK mutation.

## Background

Soft tissue sarcomas (STS) are a group of rare, heterogeneous mesenchymal cancers that include around 50 different histological types of cancers arising from extraskeletal soft tissues. STS represent approximately 1% of all adult tumours. Inflammatory myofibroblastic tumour (IMT), also called inflammatory pseudotumour [1], is an even rarer STS, characterized by local aggressiveness and low metastatic potential, consisting of a cluster of fusiform cells on a myxoid base with lymphoplasmacytic infiltrates [2, 3]. IMT may arise from different organs, being the lung the most frequent site, followed by omentum, mesentery and retroperitoneum [2]. Gynaecological IMT is an extremely rare entity.

Herein, we report the case of a 47-year-old patient with IMT of cervical origin managed in our institution’s gynaecological multidisciplinary team. During the patient’s hospital course, informed consent was obtained from the patient for the presentation of her case along with the associated medical imaging.

## Case presentation

**Fig. 1:**
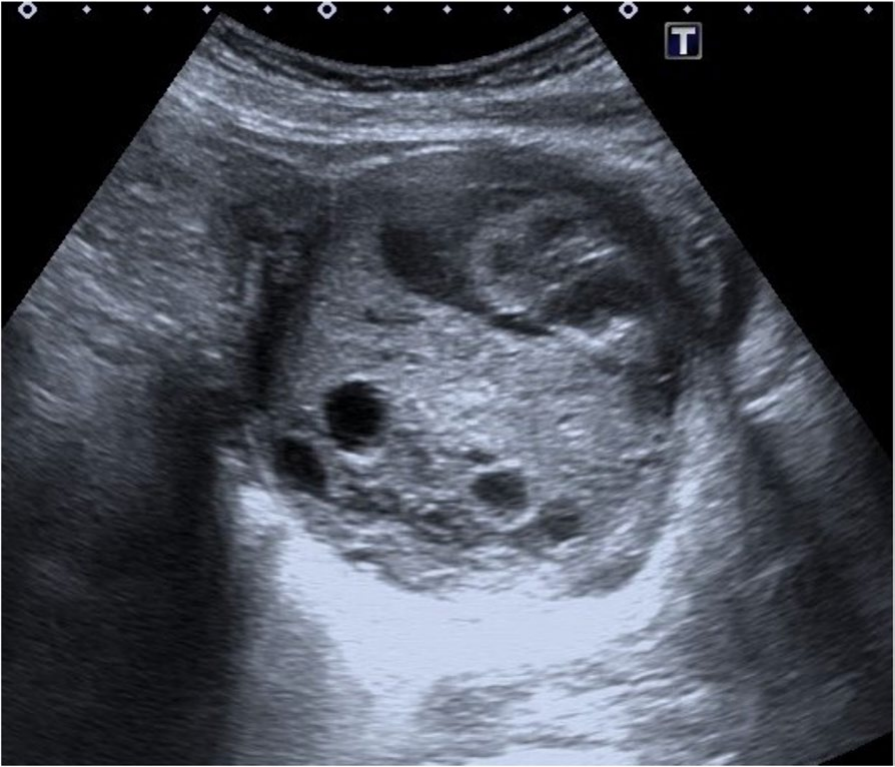
Transabdominal ultrasound. Large pelvic mass located below the corpus uteri. The lesion is heterogeneous with echogenic and anechoic areas, showing good US through-transmission which resembles fibroids with hyaline or cystic degeneration. Some Doppler signal was detected on the echogenic parts of the mass (not shown)

**Fig. 2:**
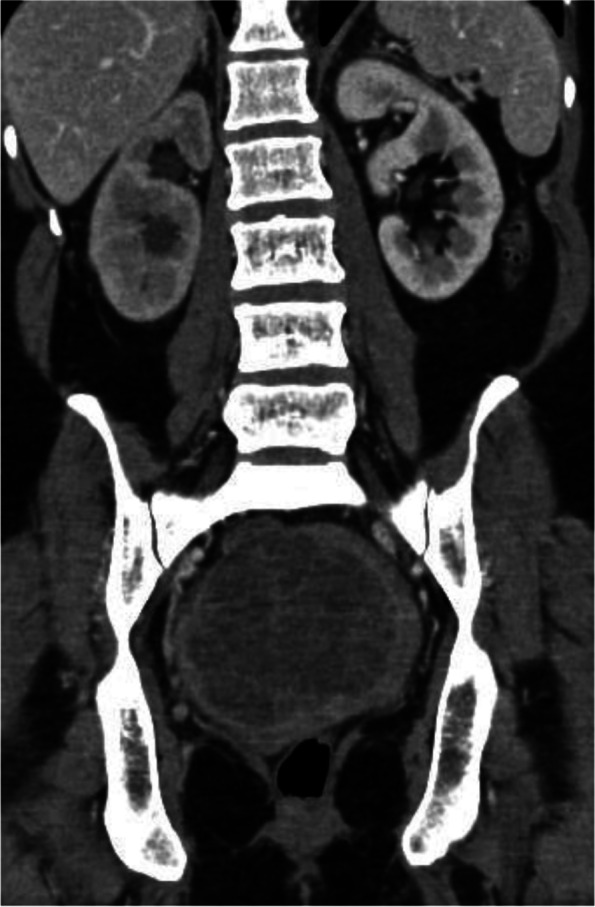
Coronal reformatted CT image with IV contrast in a portal phase. Large pelvic mass, well-defined, predominantly hypoattenuating, with many thin septa and some enhancing areas in the periphery and the centre of the lesion. The right kidney shows mild calyceal dilation and delayed and diminished cortical enhancement reflecting obstructive uropathy

**Fig. 3:**
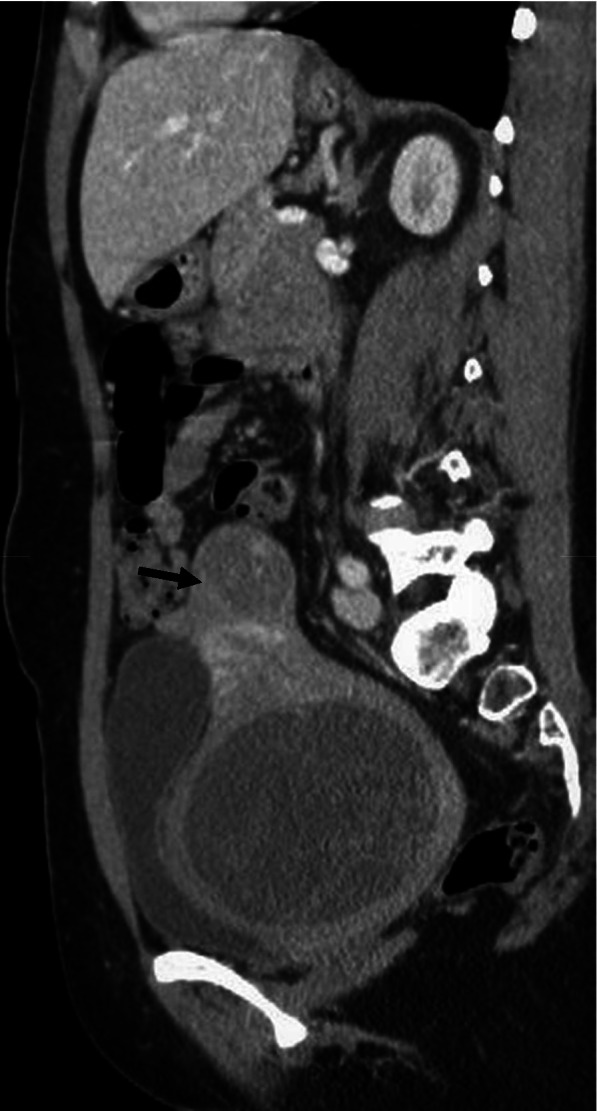
Sagittal reformatted CT image (iv contrast, portal phase). Large hypoattenuating well delineated mass located in the cervicovaginal area. There is a subserosal leiomyoma on the uterine fundus (arrowhead) and the endometrial cavity is not dilated. Bladder and rectal wall are not infiltrated by the mass

**Fig. 4:**
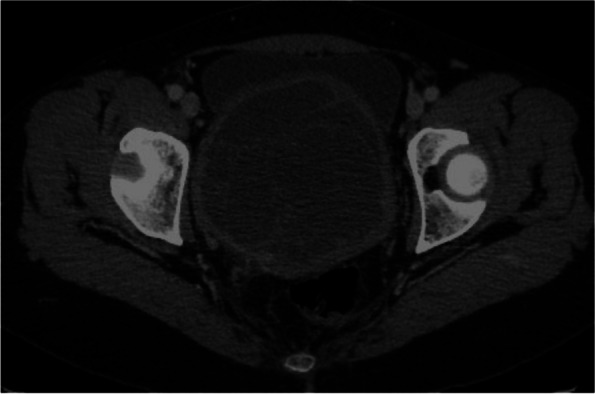
Axial CT image (IV contrast, portal phase). A large mass occupying the central part of the pelvis. It is a predominantly hypoattenuating mass with many thin septations and a thick well-delineated “capsule”. No invasion of the bladder or rectal wall was detected. No enlarged lymph nodes were present

A 47-year-old female, with no relevant medical history and no prior pregnancies, was referred to the gynaecologist for abdominal pain and dyspareunia. On gynaecological examination, we found a large gummy mass of likely uterine origin, closely attached to the vaginal wall, occupying the pelvis. The transvaginal ultrasound showed a large solid-cystic mass, with regular borders and some Doppler signal occupying the entire Douglas pouch (Fig. 1). A contrast-enhanced computerized tomography (CT) scan of the abdomen and pelvis was performed confirming a rounded 10 cm low attenuation pelvic mass predominately cystic with multiple thin septa, with an uncertain origin (uterine cervix or vagina). Despite the size of the mass, it was apparently non-infiltrative, protruding into the bladder, the rectum and the lower vagina with only a slight delay on the right renal enhancement (Figs. 2, 3, and 4). A 4-cm solid uterine mass compatible with subserous leiomyoma was revealed on the posterior surface of the uterus.

**Fig. 5:**
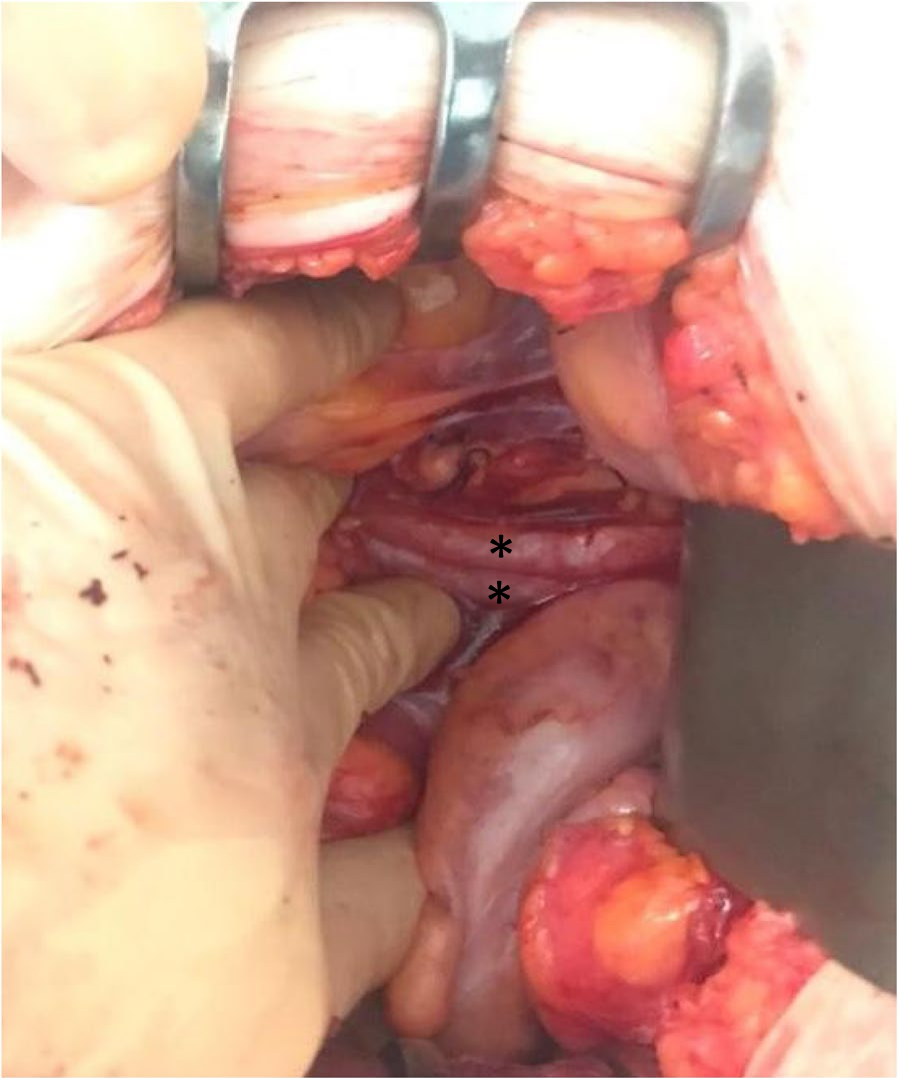
Image showing a double ureteral system on the left side

**Fig. 6:**
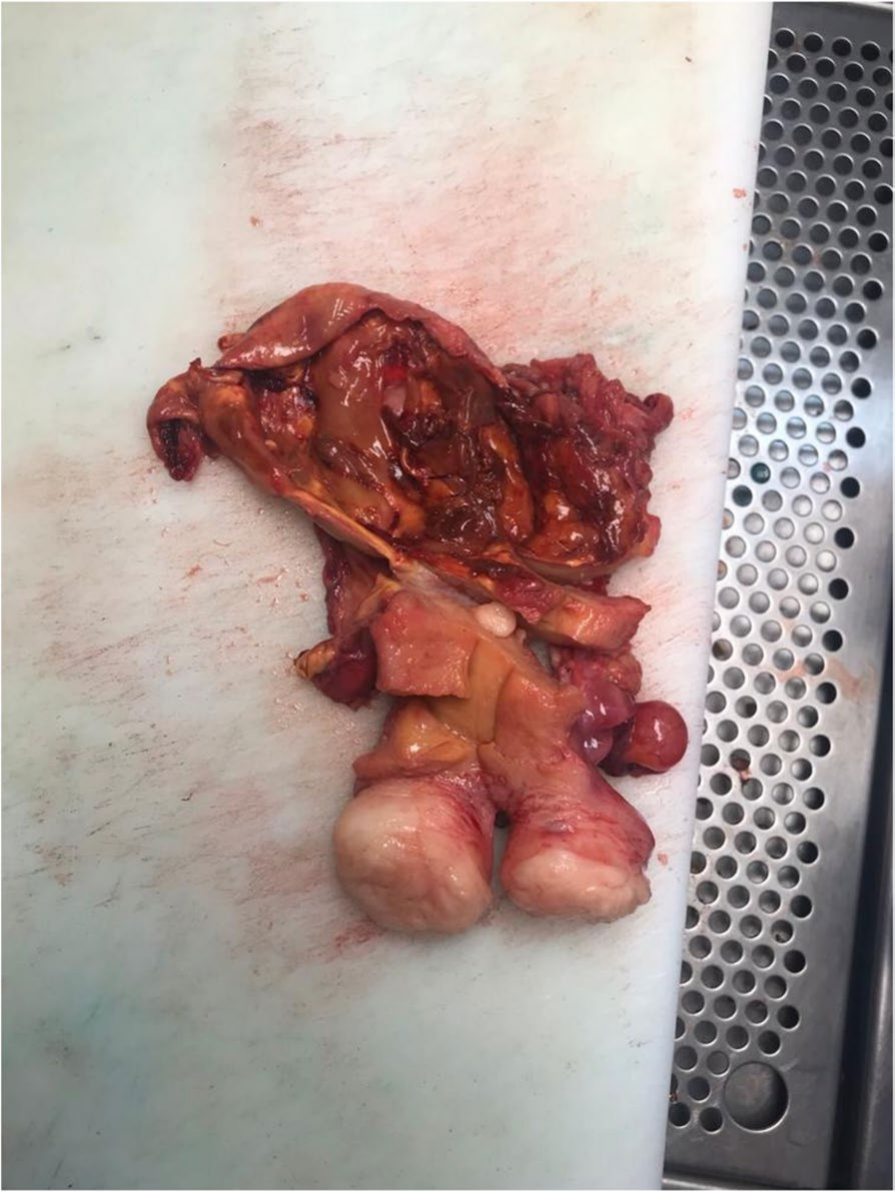
Surgical specimen: uterus opened sagittally, cystic formation cranial to the uterus, open and showing its irregular and pseudopapillary interior

**Fig. 7:**
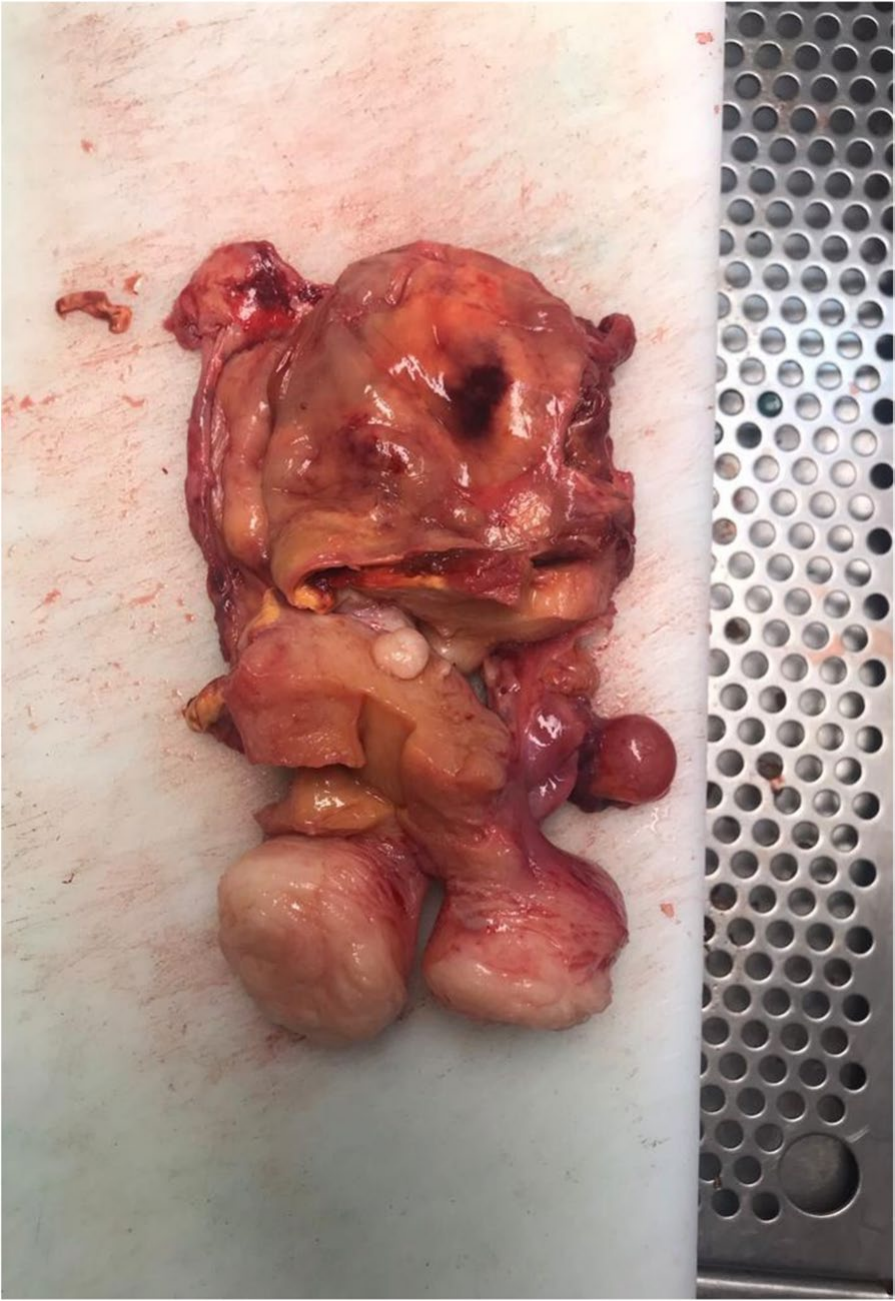
Surgical specimen: internal part of the regular tumour, uniform and fibrous wall

Based on these findings, the gynaecology oncology board agreed on resection of the pelvic mass, suspecting malignant disease. On June 5, 2019, we performed laparotomy. We found the tumour was a 10 × 15 × 12 cm cystic cervicovaginal-retrovesical mass with a whitish and smooth surface. The uterus was small with small intramuscular and subserous fibroids. The macroscopic appearance of both adnexa was normal. The tumour was intactly excised in the surgical procedure that consisted of hysterectomy, bilateral adnexectomy and paravaginal resection of the mass (Figs. 5, 6, and 7).

**Fig. 8:**
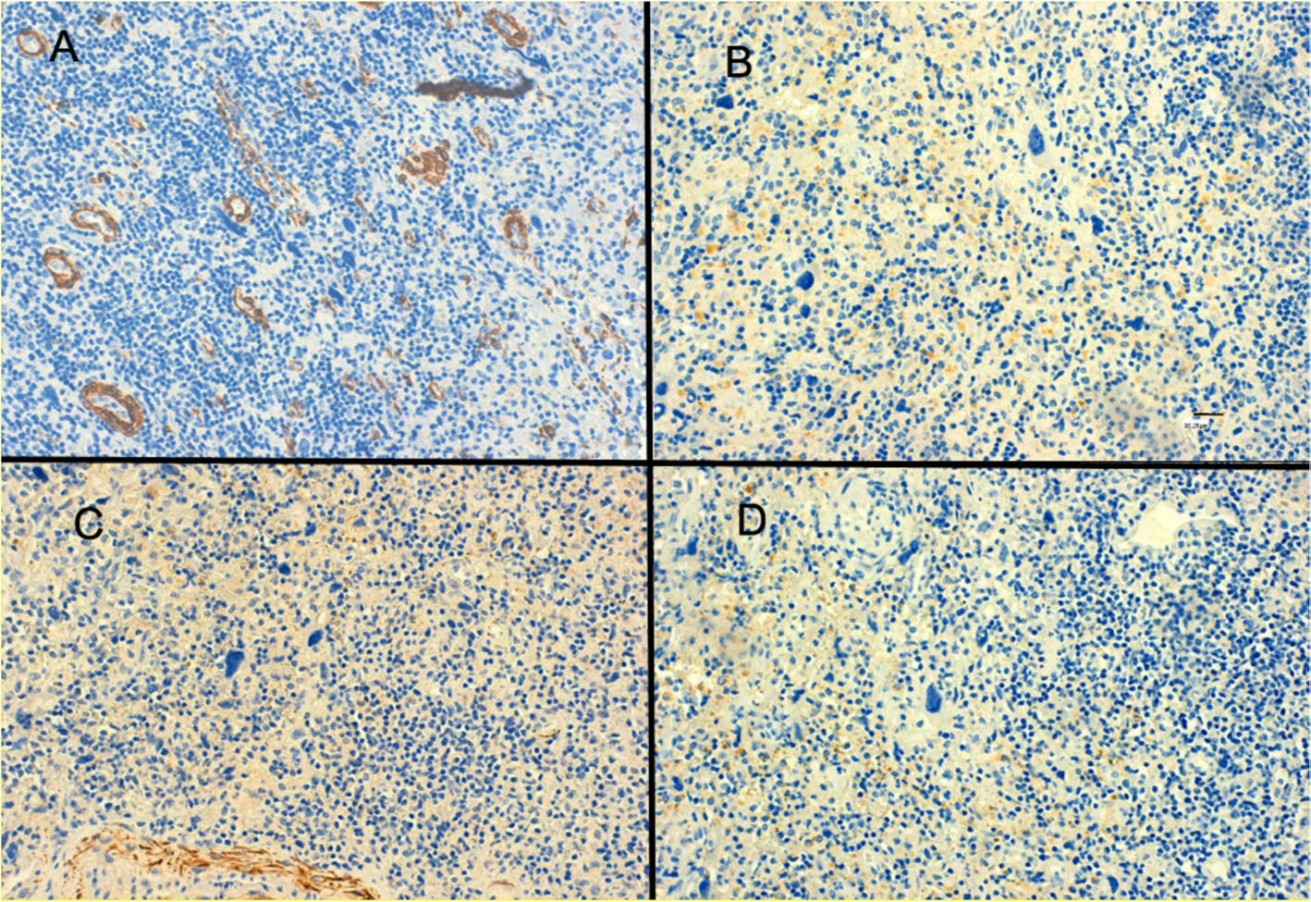
**A** Immunonegativity smooth muscle actin (original magnification ×20). **B** Immunonegativity ALK (original magnification × 20). **C** Immunonegativity desmin (original magnification × 20). **D** Immunonegativity CD10 (original magnification × 20)

**Fig. 9:**
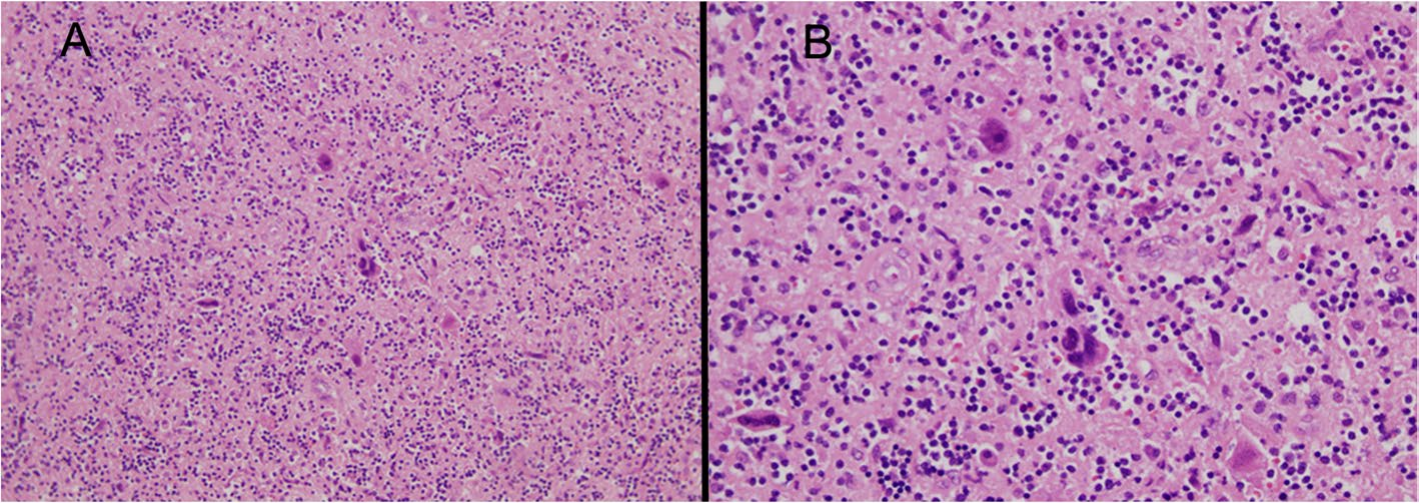
**A** Tumour cells epithelioid with marked nuclear atypia and lymphoplasmacytic infiltrate (H-E 100 x approximately). **B** Epithelioid cells with marked atypia. Nuclei are vesicular and prominent nucleoli. (H-E 400 x approximately)

The histological report confirmed large cells of poorly defined cytoplasm with anisokaryosis with focal fusiform morphology, surrounded by abundant inflammatory cells. The immunohistochemical (IHC) analysis revealed no expression for CK-AE1/AE3, EMA, ALK, CD-34, CD-45, CD-68, actin, desmin, myogenin, CD10, myoD1 and S-100 on the large cell’s component (Fig. 8), whereas expression was detected for CD-45, CD-3, CD-68 and focally for CD-20 and CD-138 on the inflammatory component. In addition, the Ki-67 labelling index was low, with less than 1% positive cells in the large cell’s component. Finally, the IHC analysis was in line with inflammatory myofibroblastic tumour (Fig. 9). No anaplastic lymphoma kinase (ALK) rearrangement nor deletion was detected on fluorescence in situ hybridization (FISH) analysis despite several attempts. The most plausible reason of ALK negative in the sample was that the tumour blocks accounted with a very few number of tumour cells. There were not enough tumour cells for FISH technique. In June 2020, a comprehensive genomic profiling FoundationOne® test (Roche) was used to analyse genomic changes in the primary tumour; unfortunately, the molecular analysis could not be applied due to lack of the minimum number of cells required in the paraffin sample.

Following surgery, a whole-body positron emission tomography-computed tomography (PET-CT) scan performed showed no evidence of malignant disease. The patient has not received any adjuvant treatment to date. Continuous patient follow-up showed no evidence of relapse to date. The last follow-up was in May 2021, with no evidence of disease for 19 months postoperatively.

## Discussion

The reported case illustrates an unusual origin of gynaecological IMT. Gynaecological IMT has been previously described in the scientific literature since 1987 [4], being uterine corpus the primary site of the gynaecological IMT cases reported to date in the English-language literature (Table 1) [3–14]. To the best of our knowledge, this is the first report of a patient diagnosed with IMT of cervix origin treated with surgery with a follow-up of 2 years with no recurrent disease.

**Table 1:** Prior case reports (in chronological order) on gynaecological IMT patients

First author, publication, year	Patient’s age at diagnosis	Symptoms	Primary tumour	Tumour size (cm)	Treatment	Follow-up period
Gilks [4], 1987	6 30	Abdominal pain Incidental	Myometrial mass Myometrial mass	12.5 4.5	Hysterectomy Hysterectomy	5 years 4.5 years
Azuno [5], 2003	60	Fever and weight loss	Myometrial mass	5 × 5	Simple hysterectomy	4 years
Rabban [3], 2005	14 25 38 45 46	Fever, weight loss Menorrhagia Pain, menorrhagia Fatigue Menorrhagia	Myometrial mass Polyp from lower uterine wall Polyp from lower uterine wall Multinodular mass in myometrium Polyp from lower uterine wall	NA 5 1 9 3	Hysterectomy Hysteroscopic resection Hysteroscopic resection Hysterectomy Hysterectomy	LTFU LTFU 3 years 2.5 years 1.5 years
Shintaku [6], 2006	63	Lower abdominal distension	Myometrial mass (posterior wall)	11	Total abdominal hysterectomy and bilateral salpingo-oophorectomy	8 months
Olgan [7], 2011	28	Abdominal pain, menorrhagia	Polyp filling the uterine cavity	2	Hysteroscopic resection	1 year
Kushnir [8], 2013	30	Abdominal pain, postcoital bleeding	Uterus, cervix, bilateral fallopian tubes and ovaries, pelvic wall, bladder and rectosigmoid peritoneum, and parametrium	6.3	Modified radical hysterectomy, bilateral salpingo-oophorectomy, resection of pelvic wall mass, pelvic peritoneum, rectosigmoid implants, bladder peritoneum	6 months
Parra-Hernan [9], 2015	29 36 37 39 40 43 45 46 55 73	Bleeding Palpable mass Bleeding Palpable mass Palpable mass Palpable mass Pain, bleeding Bleeding Palpable mass Palpable mass	Uterine fundus Lower uterine segment Uterine fundus Cervix, uterine fundus Lower uterine segment Cervix Uterine fundus Uterine fundus Uterine fundus Uterine fundus	4.2 1.3 1.5 7 7 11 5.5 NA 19.5 10.5	Hysterectomy Tumoural excision Hysterectomy Hysterectomy Hysterectomy Hysterectomy Hysterectomy Endometrial curettage Hysterectomy Hysterectomy	1 year NA 3 years 2 months 6 months NA 16 months NA 2 years NA
Fraggetta [10], 2015	10	Menorrhagia, pelvic pain	Cervical polypoid mass, two pelvic lymph nodes	8	Hysterectomy and pelvic lymphadenectomy	Almost 2 years
Subbiah [11], 2015	50’s	Pelvic discomfort	1. Uterine mass ➔ 2. Pelvis wall, peritoneum, bladder, and peritoneal cul-de-sac metastases ➔ 3. Liver and vaginal metastases	NA	1. Morcellation ➔ 2. Bilateral salpingo-oophorectomy, pelvic lymphadenectomy, and omentectomy ➔ 3. Crizotinib, pazopanib	3 years and 9 months
Mandato [12], 2017	36	Uterine bleeding	Myometrial mass, intrauterine polypoid mass	3	Total hysterectomy	6 months
Takhashi [13], 2018	44	Anaemia	Myometrial mass	7.4	Total hysterectomy	2.5 years
Zarei [14], 2020	29	Uterine bleeding	Myometrial mass	3.5	Radical hysterectomy	1 year
Current case, 2021	47	Abdominal pain, dyspareunia	Cervix	10	Total Hysterectomy and bilateral oophorectomy	2 years

*NA* not available, *LTFU* lost to follow-up

The current prevalence of IMT of gynaecological origin is difficult to be established due to the low number of published cases and the changing nomenclature and definition throughout the years (plasma cell granuloma, myofibrohistiocytic proliferation, inflammatory pseudotumour) [15]. IMT is classified as a mesenchymal neoplasia of intermediate malignant potential. The majority of cases are locally aggressive, but distant metastases at presentation and recurrences have been reported in up to 25% of patients [2, 15].

IMT’s aetiology remains unknown. Association with previous trauma, infections or inflammatory processes have been suggested [16]. Myofibroblasts are cells derived from the differentiation of dermal fibroblasts, initiated by TGF-Beta signalling pathway, which is activated for instance, in the process of wound healing. In fact, it has been shown that myofibroblasts activity is a crucial factor for scar development, which can lead to organ injury and fibrosis [17–20]. Advances in the understanding of this disease have been achieved within last years with the description of mutations in the gene that encodes for ALK at 2p23 in up to 50% of cases [21].

IMT has been described in several locations, being the lung the most frequent site, followed by omentum, mesentery and retroperitoneum [2]. The diagnosis of an inflammatory myofibroblastic tumour is extremely rare in the female genital tract [12, 22]. The average age at diagnosis is 40 years, whereas extrauterine IMT is more commonly diagnosed in children and adolescents. Cervical IMT is, therefore, a very uncommon tumour and, consequently, it is diagnosed at the histopathology analysis of the surgical specimen or biopsy performed with the clinical suspicion of other mesenchymal neoplasia as uterine leiomyomas or leiomyosarcomas [3].

IMT clinical presentation usually consists of local symptoms secondary to the mass effect and systemic symptoms such as fever, weight loss and elevation of acute phase reactants, probably related to the elevation of IL-6 levels [2, 15]. IMT has not specific radiological features. The average size at diagnosis is around 6 cm in diameter [22], in contrast with the larger size of the lesion described in our case, presenting with a mass 10 cm associating mild ureteral dilation. The radiological presentation is variable, depending on the location and the histological components of the lesion, thus modifying the contrast uptake, attenuation or Doppler signal visible at different imaging examinations [23].

Histologically, three basic patterns have been described in IMT. The myxoid pattern is the most common. It is hypocellular, and it is characterized by loosely arranged plump to spindle cells in an oedematous or myxoid stroma and a mixed inflammatory infiltrate. The second pattern consists of hypercellular regions of fascicular arrangement of spindle cells with elongated plump nuclei resembling smooth muscle cells. The third pattern counts with areas of hyalinised, sparsely cellular collagen. Mitotic activity and necrosis are rarely seen. The inflammatory infiltrate is commonly lymphoplasmacytic [2, 3, 24, 25]. The presence of aneuploidy and loss of expression of p53 has been related to more aggressive behaviour. Approximately half of the IMT have a translocation that activates ALK gene located at 2p23; this mutation is more frequently reported in tumours of gynaecological origin, even as high as 80–100% depending on the series [16, 24–26].

ALK status determination is important in this entity. A phase II study investigating the activity and safety of the ALK tyrosine kinase inhibitor, crizotinib, has recently been published showing a benefit in terms of objective response in ALK-positive IMT. This study included patients with locally advanced or metastatic IMT. Crizotinib showed benefit mainly in patients carrying ALK mutations, although the subgroup of patients without ALK mutations also showed a minor benefit [27, 28]. In our case, ALK analysis was negative by immunochemistry and FISH, and no further molecular analysis could be performed due to lack of optimal amount of tumour cells in the tumour sample for this analysis.

The most common treatment for these tumours consists of surgical excision. In gynaecological tumours, the most frequent intervention is hysterectomy, followed by resection by hysteroscopy when presenting as an intrauterine mass [7]. The relapse rate in resected pulmonary IMT is low, about 2%, while in extra-thoracic locations reaches 25%. It is recommended to perform a close follow-up at least the first years after surgery. In patients with bulky or metastatic disease (mainly lung o brain lesions) that are poor candidates for surgical treatment, therapeutic approaches have been proposed with chemotherapy and radiotherapy as well as with non-steroidal anti-inflammatories and corticosteroids [7]. IMT spontaneous remission is unlikely with only a case reported to date in English literature of these phenomena in IMT of uterine origin [29].

## Conclusion

The case reported here is unique considering the fact that IMT of cervical origin is extremely uncommon, treated with total hysterectomy and currently on surveillance with no evidence of disease relapse. IMT is a rare mesenchymal tumour and cases located on the uterine cervix are anecdotical.

## Data Availability

Not applicable.

## Abbreviations

IMT: Inflammatory myofibroblastic tumour
CT: Computerized tomography
IHC`: Immunochemistry
ALK: Anaplastic lymphoma kinase
FISH: Fluorescence in situ hybridization
PET-CT: Positron emission tomography-computed tomography
